# Chemical Composition of Natural Hydrolates and Their Antimicrobial Activity on *Arcobacter*-Like Cells in Comparison with Other Microorganisms

**DOI:** 10.3390/molecules25235654

**Published:** 2020-11-30

**Authors:** David Šilha, Karolína Švarcová, Tomáš Bajer, Karel Královec, Eliška Tesařová, Kristýna Moučková, Marcela Pejchalová, Petra Bajerová

**Affiliations:** 1 Department of Biological and Biochemical Sciences, Faculty of Chemical Technology, University of Pardubice, Studentská 573, 532 10 Pardubice, Czech Republic; karolina.svarcova@student.upce.cz (K.Š.); karel.kralovec@upce.cz (K.K.); eliska.tesarova@student.upce.cz (E.T.); marcela.pejchalova@upce.cz (M.P.); 2 Department of Analytical Chemistry, Faculty of Chemical Technology, University of Pardubice, Studentská 573, 532 10 Pardubice, Czech Republic; tomas.bajer@upce.cz (T.B.); kristyna.mouckova@student.upce.cz (K.M.)

**Keywords:** *Lavandula angustifolia*, *Syzygium aromaticum*, *Foeniculum vulgare*, *Laurus nobilis*, hydrolates, distillation, *Arcobacter*-like bacteria, antimicrobial activity, biofilm formation, gas chromatography

## Abstract

Hydrolates obtained via the hydrodistillation and steam distillation of *Lavandula* *angustifolia* Mill., *Syzygium* *aromaticum* L., *Foeniculum* *vulgare* Mill., and *Laurus* *nobilis* L. were analyzed by gas chromatography with flame ionization detector (GC-FID) and gas chromatography coupled to mass spectrometry (GC-MS). Additionally, the hydrolates were evaluated for antimicrobial activity (disk-diffusion and microdilution method), influence on biofilm formation (Christensen method) and cytotoxicity of concentrated hydrolates against human cell lines (A549) by xCELLigence system. Using chemical analysis, 48, 9, 13 and 33 different components were detected in lavender, clove, fennel and laurel hydrolates, respectively. Lavender hydrolate contained the largest proportion of 1,8-cineol, linalool furanoxide, and linalool. The main components of laurel hydrolate were 1,8-cineol, 4-terpineol and α-terpineol. Fenchone and estragole were the most abundant in fennel hydrolate, and eugenol and eugenyl acetate in clove hydrolate. Concentrated hydrolates showed significant antimicrobial activity. Clove hydrolate was among the most antimicrobially active agents, most preferably against *C*. *albicans*, with an inhibition zone up to 23.5 mm. Moreover, concentrated hydrolates did not show any cytotoxic effect again8 st human A549 cells. In the presence of the non-concentrated hydrolates, significantly reduced biofilm formation was observed; however, with concentrated clove hydrolate, there was an increase in biofilm formation, e.g., of *A*. *thereius*, *A*. *lanthieri*, and *A*. *butzleri*. Research shows new findings about hydrolates that may be important in natural medicine or for preservation purposes.

## 1. Introduction

Essential oils (EOs) are an example of highly volatile and water-insoluble plant substances. The chemical composition of EOs is highly complex. The main components include monoterpenes and sesquiterpenes [1,2,3], and they may also contain aldehydes, alcohols, ketones, acids, esters, or phenylpropanoids [4,5,6,7,8]. EOs can be extracted from plants by several methods. One of the oldest and most used methods is distillation [2]. Aqueous solutions obtained as by-products of the distillation of EOs are known as hydrolates or hydrosols [9,10]. Hydrolates have an intense herbal aroma and consist mainly of water and plant material components. Overall, they contain many bioactive hydrophilic substances [11]. Hydrolates are less described in the literature than EOs. However, it is known that the difference in the composition of a hydrolate from that of an EO is mainly quantitative [6].

Hydrolates also generally contain fewer components, or components at lower concentrations than essential oils, but they still exhibit interesting antimicrobial effects against the microorganisms of interest. Thus, they can be used in medicine or also as a potential preservative in the food industry [11,12,13,14,15]. The antimicrobial properties of substances are often influenced by the agronomic area of cultivation and processing of the plant, which affect the proportion of active ingredients [16,17]. Given the increasing resistance of microorganisms, it is appropriate to pay attention to the antimicrobial effects of natural substances, even in synergistic combination with certain antibiotics [11,18,19]. The main problem is the resistance of a microorganism with pathogenic potential. Such microorganisms include, but are not limited to, the *Arcobacter*-like bacteria. Arcobacters are Gram-negative, slender, spiral-shaped rods, and belong to the family *Arcobacteraceae* [20]. The association of arcobacters with human disease has been demonstrated for *A*. *butzleri*, *A*. *cryaerophilus*, *A*. *skirrowii* and *A*. *thereius* species [21,22]. For these bacteria, survival in the form of planktonic cells or in a biofilm is essential, as they can thus better colonize hosts or contaminate food factory environments [23,24]. The major routes of arcobacters transmission to humans include consumption of contaminated foods of animal origin and drinking non-treated water [25,26].

The aim of this study was to broaden the current knowledge of the chemical composition and antimicrobial activity of hydrolates obtained from lavender (*Lavandula angustifolia* Mill.), clove (*Syzygium aromaticum* L.), fennel (*Foeniculum vulgare* Mill.), and bay leaves (*Laurus nobilis* L.). All of these natural matrices are widely available, but to date studies have not looked at testing hydrolates obtained from these matrices. Individual hydrolates obtained by distillation were subjected to chemical analysis by gas chromatography with flame ionization detector (GC-FID) and gas chromatography coupled to mass spectrometry (GC-MS). The antimicrobial activity of the obtained hydrolates was determined by the disc diffusion method, and minimal inhibitory/bactericidal concentration (MIC/MBC) was evaluated by microdilution method. The biofilm formation of microorganisms in the presence of the tested hydrolates was followed by a modified Christensen method in microtiter plates. As far as we know, the effect of natural hydrolates on *Arcobacter*-like bacteria and others has not yet been described, although resistance to commonly used antibiotics observed among *Arcobacter*-like cells emphasizes the importance of research in this area of natural compounds [27,28,29,30,31].

## 2. Results and Discussion

### 2.1. Chemical Composition of Hydrolates

Table 1 lists some results of the analysis of concentrated hydrolates obtained by steam distillation (H_SD_SPE_) and hydrodistillation (H_HD_SPE_). The analyzed hydrolates, especially from lavender and laurel, were rich in a number of compounds. When comparing the results with respect to the hydrolate obtained by hydrodistillation (H_HD_) and steam distillation (H_SD_), more compounds were detected in the extracts from steam distillation (SD) hydrolates. This was probably caused by the more favorable conditions during steam distillation. During hydrodistillation, the plant material is in contact with boiling water throughout the distillation, and some of the volatile compounds may be converted (via oxidative reactions, polymerization and hydrolysis) into different substances [32]. The content of individual compounds is expressed as a percentage of the total peak area in the chromatograms obtained by the GC-FID analysis of H_SD_SPE_ extracts and H_HD_SPE_ extracts, so these are approximate values.

#### 2.1.1. Lavender Hydrolates

In total, 48 compounds (Table 2) were identified by mass spectrometry and by comparing the calculated retention indexes with the literature data. The identified compounds amounted to 93.2% in the H_HD_SPE_ extracts and 90.0% in the H_SD_SPE_ extracts. The major group of identified compounds was oxidized monoterpenes (34 identified compounds that accounted for 86.5% in the H_HD_SPE_ extract and 82.3% in the H_SD_SPE_ extract). The biggest difference in the chemical compositions of both extracts from lavender hydrolates was in the content of linalool (23.2% in the H_HD_SPE_, 7.9% in the H_SD_SPE_). Linalool is one of the major compounds of lavender oils. Another major compound in lavender oils is linalyl acetate. The decomposition of linalyl acetate to linalool due to hydrolysis has been reported [33], and could be the main cause of the higher content of linalool in the hydrolate obtained by hydrodistillation (HD) due to the more unfavorable conditions in HD than in SD. A relatively high difference was observed in the content of coumarins (namely coumarin and 7-methoxycoumarin; H_SD_SPE_ 3.8% in total, H_HD_SPE_ 0.9% in total), and also in the proportion of linalool derivatives (mainly furanoxides and pyranoxides; H_SD_SPE_ 23.5% in total, H_HD_SPE_ 15.7% in total). The second most abundant compound in the extracts was 1,8-cineol, the content of which was similar (20.6% in the H_SD_SPE_ extract, 19.5% in the H_HD_SPE_ extract).

#### 2.1.2. Bay Leaves Hydrolates

In total, 33 compounds were identified in both types of hydrolates (Table 3). The identified compounds amounted to 78.0% in the H_HD_SPE_ extracts and 79.4% in the H_SD_SPE_ extracts. The composition of both hydrolates was similar with respect to the identified compounds. Most of the identified compounds were oxidized monoterpenes (22 compounds accounted for 72.1% in the H_SD_SPE_ extracts and 73.2% in the H_HD_SPE_ extracts). The dominant compound in both types of extracts was 1,8-cineol (56.4% in the H_SD_SPE_ extract, 54.1% in the H_HD_SPE_ extract). Additionally, the EO of this matrix contains especially high levels of 1,8-cineol, linalool and R-terpinylacetate, as well as benzene compounds (eugenol, methyleugenol and elemicin) [34].

#### 2.1.3. Fennel Hydrolates

In total, 13 compounds were identified in both types of hydrolates (Table 4), and these compounds amounted to 84.1% in the H_SD_SPE_ extracts and 85.1% in the H_HD_SPE_ extracts. The main compounds in hydrolates were estragole and fenchone; both compounds formed almost 50% of the extracts. Estragole and fenchone are the major components of fennel EOs [35,36,37]. Furthermore, estragole was characterized as one of the key odorants in fennel EOs [38]. Another major constituent (often the most abundant compound) of fennel EOs, (E)-anethole, was not identified.

#### 2.1.4. Clove Hydrolates

In total, 9 identified compounds (Table 5) amounted to 99.3% in both extracts. The major group of identified compounds were phenolic derivatives, namely eugenol (E, 92.7% in H_HD_SPE_ extract, 89.1% in H_SD_SPE_ extract) and eugenyl acetate (EA, 9.4% in H_SD_SPE_ extract, 5.6% in H_HD_SPE_ extract). The difference in the E/EA ratio is given by the more unfavorable conditions during HD than during SD. Eugenyl acetate is hydrolyzed to form eugenol. It is similar to linalyl acetate, which is hydrolyzed during the hydrodistillation of lavender oil (see 2.1.1).

### 2.2. Antimicrobial Activity of Hydrolates

The antimicrobial activities of eight samples of non-concentrated and eight samples of concentrated hydrolates from *Lavandula angustifolia* Mill., *Syzygium aromaticum* L., *Foeniculum vulgare* Mill., and *Laurus nobilis* L. against eight strains of *Arcobacter*-like bacteria, and further against *Staphylococcus aureus* CCM 4223, *Enterococcus faecalis* CCM 4224, *Pseudomonas aeruginosa* CCM 3955, *Escherichia coli* CCM 3954 and the yeast *Candida albicans* CCM 8186, are presented in Table 6 and Table 7.

#### 2.2.1. Non-Concentrated Hydrolate (H_HD_, H_SD_)

For most of the tested hydrolates H_HD_ and H_SD_, no antimicrobial activity against the observed microorganisms was recorded. For this reason, the data are not presented in the form of a table. Most of the studied arcobacters were not suppressed by non-concentrated hydrolates at all. However, a very weak antimicrobial activity of clove hydrolate was reported just against *A*. *thereius* LMG 24488 (H_HD_, inhibition zone 6.5 ± 0.3 mm; H_SD_, inhibition zone 6.8 ± 0.4 mm). None of the tested arcobacters were suppressed by the hydrolates obtained by the distillation of lavender, fennel and laurel.

According to our results, weak antimicrobial activity on other Gram-negative (*Ps*. *aeruginosa*, *E*. *coli*) bacteria was observed only in the case of clove hydrolate, in the range of inhibition zones 7.0–7.3 mm. In general, Gram-negative bacteria are more resistant to, e.g., EOs than Gram-positive bacteria, mainly due to the composition of the cell wall [39]. In the case of Gram-positive (*S*. *aureus*, *Ent*. *faecalis*) bacteria, the weak antibacterial activity of all monitored hydrolates was observed. The highest antimicrobial activity on Gram-positive bacteria was observed in the case of fennel and clove hydrolates. In *S*. *aureus* CCM 4223, with fennel or clove H_HD_/H_SD_, inhibition zones of 8.0/8.0 and 7.8/8.3 mm were observed, respectively. Similar antimicrobial effects of fennel EO and extract have also been reported in earlier publications [40,41]. The *Ent*. *faecalis* CCM 4224 strain was most inhibited by bay leaf (H_HD_, 7.8 ± 1.3; H_SD_, 7.8 ± 0.5 mm), fennel (H_HD_, 7.8 ± 0.5; H_SD_, 7.8 ± 0.5 mm) and clove (H_HD_, 7.5 ± 0.5; H_SD_, 7.5 ± 0.6 mm) hydrolates. It has been previously reported that clove EO also exhibits significant inhibitory effects against *Ps*. *aeruginosa*, *S*. *aureus* and *E*. *coli* [42].

Although linalool as a pure chemical has been demonstrated [43] to be a potent antimicrobial agent against a wide range of microorganisms, except *Pseudomonas aeruginosa*, its higher percentage did not give the expected results for HD hydrolate extract in the antimicrobial assay. Coumarin and its derivatives have been evaluated as having potentially broad antimicrobial activity [44,45]. In combination with the relatively high differences in linalool derivative content, this may be one of the causes of the higher antimicrobial activity of H_SD_ extracts (see Table 6). It was previously reported that the high linalool content or its addition to an EO significantly increases its antimicrobial effects [46]. A greater antimicrobial effect of the laurel-derived hydrolate against Gram-positive bacteria has been reported in the literature [47]. However, this has not been unequivocally confirmed in our study. *C*. *albicans* CCM 8186 was not inhibited by the tested hydrolates at all.

The obtained results show that the prepared non-concentrated hydrolates do not have significant antimicrobial potential against the tested microorganisms. The antimicrobial activity of hydrolates is also significantly influenced by the method used to obtain the distilled matrices [11,13,48]. There is limited information in the literature about the antimicrobial effects of hydrolates, but many studies describe the antimicrobial activity of EOs. The greater antimicrobial activity of EOs is mainly due to the higher concentration of the main essential components. Importantly, even the hydrolates obtained by distillation still have antimicrobial activity [49], and may thus be further used, especially in their concentrated form.

#### 2.2.2. Concentrated Hydrolate (H_HD_SPE_, H_SD_SPE_)

In contrast, concentrated hydrolates exhibited significant antimicrobial activity (see Table 6 and Table 7) with inhibition zones up to 23.5 mm in diameter and minimal inhibitory (bactericidal) concentration up to 0.1%. The inhibitory effect of concentrated hydrolates may be partially due to the presence of extraction reagent (ethanol). The influence of ethanol on the resulting inhibitory activity of the concentrated hydrolates was, of course, also evaluated. The antimicrobial activity of ethanol is presented in Table 6 and Table 7; however, the inhibition zones of the pure solvent were only recorded in the range of 6.3–9 mm (MIC in the range 1.6–12.5%), depending on the strain tested.

Significant inhibition was observed in the presence of concentrated hydrolates H_HD_SPE_ and H_SD_SPE_. Overall, the minimum inhibitory concentrations of all monitored samples in the range of 0.1–6.3% were found. In the vast majority of cases, the MIC and MBC values were the same or lower. However, in general, most of the hydrolates obtained by steam distillation showed higher antimicrobial activities compared to hydrolates obtained by hydrodistillation. Both concentrated clove hydrolates exhibited the highest antimicrobial activity of all the matrices tested. Clove is a very rich source of various antimicrobials [50]. In addition, according to our results, a significant content of eugenol and eugenyl acetate was observed in clove hydrolates. The lowest minimum inhibitory concentrations, in the range of just 0.1–0.8%, were measured for clove hydrolate, and can be considered essentially the most antimicrobially effective.

Lavender H_SD_SPE_ had the strongest antimicrobial activity against *A*. *butzleri* CCUG 30484 and *A*. *thereius* LMG 24488 (13.5 ± 0.6 and 13.3 ± 0.9 mm inhibition zones, respectively). On the contrary, the growth of the *A*. *butzleri* UPa 2012/3 strain was more inhibited by lavender H_HD_SPE_ (inhibition zone 12.8 ± 0.3 mm). The antimicrobial effects of both lavender EO and hydrolate have also been previously described in the literature [7,51,52]. Overall, the most sensitive *Arcobacter*-like strain tested was *A*. *cryaerophilus* UPa 2013/13, in which an inhibition zone of 16.5 ± 0.3 mm was recorded for clove H_SD_SPE_. The *A*. *butzleri* UPa 2012/3 strain was the most sensitive to laurel and clove H_SD_SPE_ (inhibition zones 14.0 ± 0.8 and 14.5 ± 0.3 mm, respectively). The highest resistance of arcobacters was observed especially in relation to fennel hydrolate.

Both concentrated hydrolates (H_HD_SPE_ and H_SD_SPE_) were almost comparable in antimicrobial activity against Gram-positive *S*. *aureus* CCM 4223. The lowest inhibitory activities were observed with lavender-derived hydrolates, then laurel and fennel, and the greatest antimicrobial effect was determined with clove hydrolate, with inhibition zones up to 15.8 mm and MIC at 0.4%. In *Ent*. *faecalis* CCM 4224, an inhibition zone of 15.0 ± 0.1 mm was observed for fennel H_SD_SPE_. This is possibly due to the higher estragole and *p*-methoxy cinnamaldehyde content in H_SD_SPE_ compared to H_HD_SPE_ with lower antimicrobial activity. Conversely, however, fennel H_HD_SPE_ has a higher content of eugenol and eugenyl acetate, which are known to have high antimicrobial potential [53]. *E*. *coli* was most suppressed by clove hydrolates (inhibition zone 13.5 ± 0.7 and 14.0 ± 0.8 mm).

*C*. *albicans* CCM 8186 was not inhibited even by concentrated lavender hydrolates in the case of the disk diffusion test; however, the minimal inhibitory concentration was 6.3%. In contrast, the growth of *C*. *albicans* was suppressed by laurel H_SD_SPE_ (13.3 ± 0.9 mm inhibition zone; MIC 0.4%). It has been previously reported that laurel hydrolates have significant antifungal activity [54]. However, clove H_SD_SPE_ was the most effective against *C*. *albicans* CCM 8186 yeast (inhibition zone 23.5 ± 0.7 mm; MIC 1.6%). Antimicrobial and antifungal effects have already been described in eugenol, the main aromatic component of cloves [8,53,55,56]. The antimicrobial effect of clove hydrolate appears to be supported, inter alia, by the eugenyl acetate content, which is higher than in the H_SD_SPE_, and in which a higher antimicrobial effect was also observed compared to the H_HD_SPE_. It has previously been reported in the literature that the incorporation of acetate into a molecule may increase the antimicrobial effect [57].

Table 8 shows, for comparison, the inhibitory effects of common antimicrobials against the microorganisms involved in this study. Based on the comparison of the inhibition zones of antibiotics/antifungals and the monitored samples (Table 6 and Table 7), it is evident that the antimicrobial effect of antibiotics is higher. However, it is also necessary to look for suitable alternatives to antimicrobial substances in purely natural materials [58]. The EOs, as natural extracts, and the hydrolates obtained as by-products (with low adverse effects) may also become reliable alternatives in antimicrobial therapy.

### 2.3. Biofilm Activity of Selected Microorganisms in the Presence of Hydrolate

**Figure 1:**
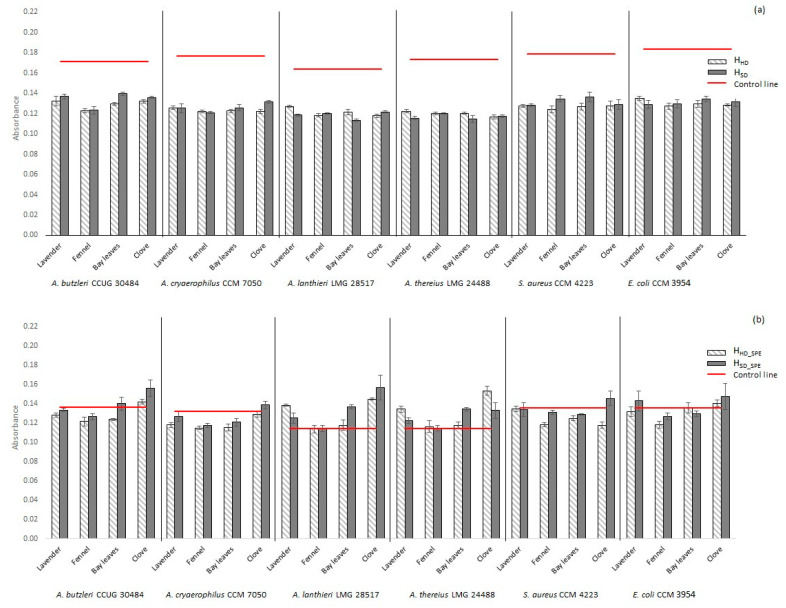
Biofilm formation in the presence of non-concentrated hydrolates (**a**) and concentrated hydrolates (**b**). Bars represent standard deviation, *n* = 3. H_HD_—hydrolate obtained by hydrodistillation; H_SD_—hydrolate obtained by steam distillation; H_HD_SPE_—hydrolate obtained by hydrodistillation and 50× concentrated using solid phase extraction (SPE); H_SD_SPE_—hydrolate obtained by steam distillation and 50× concentrated using SPE. Red lines—biofilm formation of strains in water (**a**), biofilm formation of strains in extraction solvent (**b**); values above/below the red lines—increased/reduced biofilm formation due to hydrolate presence.

The characterization of new potential antimicrobial agents is important, inter alia, in terms of the increasing resistance of microorganisms. In this respect, the inhibition of not only planktonic cells but also biofilm formation is often important [59]. The bacteria in a biofilm are more resistant and are more difficult to remove from surfaces than planktonic cells [60,61]. The biofilm formations of *A*. *butzleri* CCUG 30484, *A*. *cryaerophilus* CCM 7050, *A*. *lanthieri* LMG 28517, *A*. *thereius* LMG 24488, *S*. *aureus* CCM 4223 and *E*. *coli* CCM 3954 were studied in the presence of prepared hydrolates before and after increasing their concentration. The results are shown in Figure 1, which shows the different values of biofilm activity that also depended on the preparation method of the given hydrolate (H_HD_ vs. H_SD_). Most of the natural concentrated hydrolates obtained by hydrodistillation showed a higher antibiofilm effect (see Figure 1b). However, a higher antimicrobial effect on planktonic cells was observed for hydrolates obtained by steam distillation. This could probably be explained by the fact that the higher antimicrobial efficacy of some substances (under certain circumstances) may induce an increased biofilm formation activity of microorganisms. The effects on biofilm structures may often not be as significant as in the case of planktonic cells. This may depend, for example, on the cellular metabolic activity, and the amount of extracellular polymer matrix in the biofilm structure [62], and increased biofilm formation can also be considered when exposed to substances with a higher antimicrobial effect.

According to our preliminary testing, all microbial strains used can be considered biofilm-positive. The biofilm formation of all tested strains was reduced in the environment of the prepared hydrolates in contrast to the amount of biofilm formed in water without the presence of hydrolates (see Figure 1a, red line). Some strains exhibited a significant decrease in the amount of biofilm formed in the presence of non-concentrated hydrolates, e.g., *A*. *thereius* (by up to 35.9%) and *A*. *lanthieri* (by up to 31.0%) in the case of the H_SD_SPE_ of bay leaves hydrolate. On the other hand, a slightly reduced biofilm activity was determined in the case of, e.g., *A*. *butzleri* (by up to 18.7% in the case of the H_SD_SPE_ of bay leaves). In agreement with this, a significant antibiofilm effect of, e.g., lavender and clove EOs is described in the literature [63], which is associated with a significantly higher content of individual active substances. Previously, there has also been a mention in the literature of the antibiofilm activities of, e.g., lavender and oregano with *S*. *aureus* [63].

Significantly different results were obtained in the presence of concentrated hydrolates. In the case of concentrated hydrolates, increased biofilm formation was found in some tested strains compared to the amount of biofilm formed in the extraction solvent alone (see Figure 1b, red line). In the *A*. *thereius* LMG 24488 strain, an increase in biofilm formation was observed even in the environment of the concentrated samples (except for the fennel hydrolates). A rapid increase in biofilm activity was also observed in the clove H_SD_SPE_ environment for *A*. *lanthieri* LMG 28517 and H_HD_SPE_ for *A*. *thereius* LMG 24488. Clove H_SD_SPE_ also contains approximately twice the amount of eugenyl acetate (see Table 5), which could affect biofilm formation in these bacteria. However, the increased biofilm formation in the presence of hydrolates obtained from *Syzygium aromaticum* can be explained by the very severe stress effect. The matrix contains a considerable amount of antimicrobial agents, however the natural protective function of some microorganisms is the formation of a biofilm structure [64]. Biofilm structure is largely protected from external influences and microorganisms are more resistant. Clove hydrolate has been shown to be more antimicrobially effective against planktonic cells (Table 6), but bacteria often respond to the significant inhibitory effect of bacteria by increasing and rapid biofilm formation. Clove hydrolate contains a high proportion of eugenol (see Table 5), for which an antibiofilm effect has been previously described [65]. However, it certainly depends on the amount of the substance and also on the specific case of the microbial strain, as also stated in the publication.

However, the biofilm activity of *A*. *butzleri* CCUG 30484, *A*. *cryaerophilus* CCM 7050, and *S*. *aureus* CCM 4223 was reduced (in comparison with other strains and clove hydrolates) in the concentrated lavender, fennel, and bay leaves hydrolate environments (see Figure 1b). EO components are known to significantly reduce biofilm activity [66]. Further studies have shown that farnesol induces a decrease in biofilm production [67]. The presence of farnesol was observed in the lavender hydrolate in our study, but only at a low concentration (see Table 4), but still obviously with an interesting antibiofilm activity.

Plant EOs have an antimicrobial potential that can be used to suppress microorganisms [68]. As far as we know, the effect of natural hydrolates on biofilm formation activity has not yet been well described. According to the results of our study, hydrolates also appear to be useful in this regard. However, it has been previously reported that, for example, coriander EO and its main component (linalool) have been able to inhibit the production of a microbial biofilm in vitro [69]. Furthermore, the inhibition of the biofilm production of uropathogens (*E*. *coli*, *Ps*. *aeruginosa*, *Proteus mirabilis* and *Serratia marcescens*) by curcumin has been reported. It has also been documented that curcumin generally increases the sensitivity of some strains to antibiotics [70].

### 2.4. In Vitro Cytotoxicity of Hydrolates

**Figure 2:**
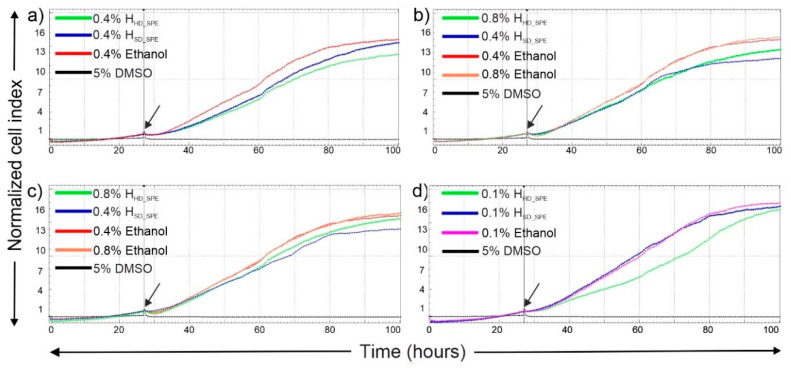
Dynamic monitoring of cytotoxic response to different concentrations of the hydrolates concentrated by SPE. A549 cells were treated with selected concentrations of (**a**) lavender, (**b**) fennel, (**c**) bay leaves and (**d**) clove concentrated hydrolates. Cells treated with 0.1%, 0.4% and 0.8% ethanol were used as vehicle control and 5% DMSO treated cells were as positive control. Arrow showing time-point of treatment. Cell index values over 72 consecutive treatment hours were normalized to the time point of treatment.

The cytotoxic level of medicinal plants or potential natural compounds must also be evaluated against host cells in the case of medical use or other applications [71]. The cytotoxicity of the hydrolates concentrated by SPE was analyzed using the xCELLigence system against the human lung carcinoma (A549) cells. It was observed that A549 cells treated with determined minimal inhibitory concentrations against microorganisms—0.4% of lavender H_HD_SPE_ and of lavender H_SD_SPE_, 0.8% of fennel H_HD_SPE_, 0.4% of fennel H_SD_SPE_, 0.8% of bay leaves H_HD_SPE_, 0.4% of bay leaves H_SD_SPE_ and 0.1% of clove H_HD_SPE_ and H_SD_SPE_—were proliferating in parallel with cells treated with vehicle control, as indicated by the increase in CI values (Figure 2). This showed that A549 cell growth was not affected, thus suggesting the negligible cytotoxicity of the concentrated hydrolates tested in this study. Several studies also describing the cytotoxicity of lavender, laurel, fennel and other extracts have been published in the past [72,73,74], and the results suggest the utility of these natural matrices for many other applications.

## 3. Materials and Methods

### 3.1. Plant Materials and Sample Preparation

Plant material was bought from the following distributors: *Lavandula angustifolia* Mill.—lavender buds, origin—Croatia (Mediate, Libchavy, Czech Republic); *Laurus nobilis* L.—bay leaves, origin—Brazil (Kasia Vera, Říčany, Czech Republic); *Syzygium aromaticum* L.—clove buds, origin—Madagascar (Kasia Vera, Říčany, Czech Republic) and *Foeniculum vulgare* Mill.—fennel seeds, origin—Czech Republic (Kasia Vera, Říčany, Czech Republic).

Prior to the analysis, samples were preserved in the dark at laboratory temperature. Before the distillation, bay leaves were cut into small pieces of ca. 0.5 × 0.5 cm. Hydrolates were obtained by hydrodistillation (HD) and steam distillation (SD). Each experiment was performed in triplicate.

#### 3.1.1. Steam Distillation

Steam distillation was performed in a steam distillation system. The distillation flask was connected to a cylindrical body containing the plant material. The hydrolate was prepared as follows: the appropriate amount of plant material was weighed into the cylindrical body. Then, 1500 mL of deionized water was placed into the 2000 mL distillation flask (water was refilled as needed) and the distillation was started. The EO and hydrolate were collected in a separator with an outlet for the hydrolate (the hydrolate was collected in a dark flask during the distillation). The distillation was finished when the amount of obtained essential oil remained constant. After distillation, the rest of the hydrolate was separated from the essential oil, put into the dark flask and stored at 4 °C. The final sample of hydrolate was obtained by mixing the hydrolates from three distillation runs.

#### 3.1.2. Hydrodistillation

Hydrodistillation was performed in the hydrodistillation system. The hydrolate was prepared as follows: the appropriate amount of plant material was weighed into a 2000 mL distillation flask, 500 mL of deionized water was added (water was replenished during the distillation) and the distillation was started. The hydrolate was collected as described in Section 3.1.1. The final sample of hydrolate was obtained by mixing the hydrolates from three distillation runs.

### 3.2. Solid Phase Extraction (SPE)

After obtaining the hydrolates (H_SD_ or H_HD_), they were 50× concentrated using Separon SGX C18 (modified octadecylsilicagel, 60 µm, 0.5 g) into a 96% ethanol. The sorbent in the column was activated with 5 mL of methanol, and finally rinsed with 10 mL of sterile distilled water. Then 100 mL of hydrolate was passed through the column using a syringe, after which the compounds were eluted from the sorbent using 2 mL of ethanol solvent. The extract obtained by SPE from hydrolates is called an H_SD_SPE_ or H_HD_SPE_ extract within the text.

### 3.3. Antimicrobial Testing

The microorganisms used in this study were as follows—*A*. *butzleri* LMG 10828, *A*. *butzleri* CCUG 30484, *A*. *butzleri* UPa 2012/3, *A*. *cryaerophilus* CCM 7050, *A*. *cryaerophilus* UPa 2013/13, *A*. *lanthieri* LMG 28517, *A*. *skirrowii* LMG 6621, *A*. *thereius* LMG 24488, *S*. *aureus* CCM 4223, *Ent*. *faecalis* CCM 4224, *Ps*. *aeruginosa* CCM 3955, *E*. *coli* CCM 3954, and *C*. *albicans* CCM 8186. Strains were purchased from the Czech Collection of Microorganisms (CCM, Brno, Czech Republic), Culture Collection University of Göteborg (CCUG, Göteborg, Sweden), and Belgian Co-ordinated Collections of Microorganisms (LMG, Ghent, Belgium), and were isolated at the University of Pardubice (UPa, Pardubice, Czech Republic).

The antimicrobial activity of hydrolates (from steam distillation and hydrodistillation) obtained from lavender, bay leaves, clove, and fennel was tested. The disc diffusion method was used to determine the susceptibility of the strain to the hydrolates. A cell suspension with turbidity according to the McFarland scale were freshly prepared corresponding to the grade 0.5 (~1.5 × 10^8^ CFU·mL^−1^). The microbial cell suspension was spread onto Mueller-Hinton agar (MHA; Himedia, India) or Sabouraud dextrose agar (Himedia, India) plates using a sterile cotton swab. Sterile discs of 6 mm in diameter (Oxoid Ltd., UK) were impregnated with 8 μL of the tested sample (or ethanol as a control). After cultivation at 30 °C (*Arcobacter*-like bacteria) or 37 °C (other microbes), the diameter of the inhibition zone was evaluated using a BACMED 6iG2 automated reader and analyzer (Aspiag, Litomyšl, Czech Republic). Commercial antimicrobial discs (Sigma-Aldrich, St. Louis, MO, USA) were used as a reference standard of antimicrobial activity—ampicillin (10 μg), ciprofloxacin (5 μg), clindamycin (2 μg), erythromycin (15 μg), fluconazole (25 μg), and tetracycline (30 μg). Each experiment was performed at least in triplicate, and the displayed inhibition zones are expressed as a mean of inhibition zones with standard deviation.

Minimal inhibitory concentration (MIC) was determined by a microdilution method in 96-well microtiter plates (SPL Life Sciences, Pocheon-si, South Korea). Hydrolates in Mueller-Hinton broth (MHB; Himedia, India) at concentrations of 0.02–50% were evaluated in a final volume of 100 µL in each well. The wells were inoculated with a microorganism suspension at a density of 1.5 × 10^6^ CFU·mL^−1^. After cultivation, MIC was detected by lack of visual turbidity. For the evaluation of minimal bactericidal concentration (MBC), the content of each well was sub-cultured on Mueller-Hinton agar or Sabouraud dextrose agar. MBC was stated as the lowest concentration of the hydrolate that completely inhibited the growth of microorganisms (99.9%) in CFU·mL^−1^. Each experiment was performed at least in triplicate, and the displayed concentrations are expressed as a mean.

### 3.4. Biofilm Formation Determination

Biofilm formation in the presence of hydrolates obtained from lavender, bay leaves, clove, and fennel was monitored in flat-bottomed microtiter plates (SPL Life Sciences, Pocheon-si, South Korea) as previously described by Silhova-Hruskova [75], with modifications. Briefly, the strain cultured in brain heart infusion broth (BHI; Himedia, India) was prepared at a final cell concentration of 10^7^ CFU·mL^−1^. After incubation at 30 °C for 24 h under aerobic conditions, the microtiter plate was washed with sterile distilled water and dried. Biofilm fixation was performed with 2% sodium acetate (15 min) and biofilm-forming cells were stained with 100 µL of 1% crystal violet (Sigma-Aldrich, St. Louis, MO, USA). After 15 min of staining, the plate was repeatedly washed and dried. Thereafter, the biofilm-associated violet was solubilized with 96% ethanol and the absorbance of the solution was measured in a new plate at 595 nm (Infinite M200, Tecan, Männedorf, Switzerland).

In addition, the influence of ethanol (used for SPE concentration of hydrolates) was monitored in order to determine the real effect of the tested hydrolates on biofilm formation, as ethanol also affects biofilm activity. The experiments were performed as above, but just in the presence of ethanol. The biofilm formation in the presence of ethanol is presented by red lines in Figure 1b.

There were 8 wells in each experiment, and the experiments were independently repeated three times. The obtained values were statistically evaluated using Excel 2010 (Microsoft, Redmond, WA, USA) and Statistica 12 (StatSoft, Tulsa, OK, USA). Extreme values were tested with the Dean–Dixon test, and all remoteness values were excluded with 95% probability. Median and standard deviations were determined from the remaining values. A possible source of error resulting from insufficient dye washing, and resulting in an increase in absorbance, was also considered, and absorbance values that were too high compared to other measured values were excluded.

### 3.5. GC-MS Analysis

GC-MS analysis of the obtained extracts was carried out with a GC 2010 Gas Chromatograph, GCMS-QP2010 Plus Mass Selective Detector (both Shimadzu, Kyoto, Japan) and Combi Pal Autosampler (CTC Analytics, AG, Zwingen, Swizerland). The GC was equipped with an SLB-5ms capillary column (30 m × 0.25 mm, 2.5 μm film thickness; Supelco, Bellefonte, PA, USA). Helium 5.0 (Linde, Praha, Czech Republic) was used as the carrier gas at a constant flow rate of 30 cm·s^−1^. The GC oven temperature was programmed as follows: initial temperature of 40 °C for 3 min, then heated up to 250 °C at 2 °C·min^−1^. The injector temperature was set to 200 °C. Then, 1 µL of extract was injected with a split ratio of 1:50. Mass spectrometry was run in electron ionization mode (EI) at 70 eV, in which ions with m/z 33–500 were scanned. Ion source and interface temperatures were set to 200 °C.

Mass spectral identification was performed by comparing mass spectra with the commercial mass spectral databases NIST’14 Mass Spectral Library and FFNSC library (GC-MS Flavor & Fragrance Natural & Synthetic Compounds Library). Experimental results of retention indices were additionally compared with published data (Adams, 2007; Linstrom and Mallard, 2001) and data from FFNSC. Retention indices were calculated according to the van den Dool and Kratz method, using a standard solution of *n*-alkanes (C8–C33) at concentrations of 100–200 µg·mL^−1^, dissolved in *n*-hexane (Restek, Bellefonte, PA, USA).

### 3.6. GC-FID Analysis

GC-FID analyses of the obtained hydrolates were carried out using a GC 2010 Gas Chromatograph with flame ionization detector (Shimadzu, Kyoto, Japan) and Autosampler Combi Pal (CTC Analytics, AG, Zwingen, Swizerland). The GC-FID conditions were the same as for the GC-MS analysis. The injector temperature was set to 200 °C and the detector temperature was set to 270 °C. Then, 1 µL of extract was injected with a split ratio of 1:50. Chemical compounds were identified using the experimental results of retention indices, which were calculated according to the van den Dool and Kratz method, using *n*-alkanes as external references. The calculated retention indices were additionally compared with the retention indices of identified compounds from mass spectra analysis.

### 3.7. In Vitro Cytotoxicity Assay

#### 3.7.1. Cell Lines

A549 cells were cultured in Minimum Essential Medium Eagle with l-glutamine and sodium bicarbonate (Sigma-Aldrich, St. Louis, MO, USA) in the presence of 10% fetal calf serum, 1 mM pyruvate, 10 mM HEPES, 50 μg/mL penicillin and 50 μg/mL streptomycin (all supplements from Life Technologies, Grand Island, NY, USA) in a humidified atmosphere containing 5% CO_2_ at 37 °C. A549 cells were purchased from the European Collection of Cell Cultures (ECACC, Salisbury, UK).

#### 3.7.2. Real-Time Cytotoxicity Assay

The cytotoxicity of the concentrated hydrolates (H_HD_SPE_ and H_SD_SPE_) was assessed against A549 cells using the xCELLigence RTCA (real-time cell analysis) SP (single-plate) system (Roche Diagnostic, Germany), allowing the label-free dynamic monitoring of cell events in real-time. The principle of the system is to monitor the changes in electrode impedance induced by the interaction between testing cells and electrodes [76,77]. The xCELLigence system was connected and tested by Resistor Plate Verification before the RTCA SP station was placed inside the incubator at 37 °C and 5% CO_2_. Background measurements were taken by adding 100 μL of appropriate medium to the wells of the E-Plate 96. A cell suspension (90 μL) at cell density of 4000 cells per well was added to each well of the E-plate 96. The A549 cell proliferation was dynamically monitored at the 30 min interval. When the cells entered the logarithmic growth phase, they were treated with 10 μL of concentrated hydrolates (H_HD_SPE_ and H_SD_SPE_) dissolved in ethanol at concentrations of 0.4% for both lavender H_HD_SPE_ and lavender H_SD_SPE_ hydrolates, 0.8% for fennel H_HD_SPE_, 0.4% for fennel H_SD_SPE_, 0.8% for bay leaves H_HD_SPE_, 0.4% bay leaves H_SD_SPE_, and 0.1% for both clove H_HD_SPE_ and clove H_SD_SPE_ in triplicates. Cells treated with 0.1%, 0.4% and 0.8% ethanol were used as the vehicle control while cells treated with 5% DMSO were used as the positive control. After 72 h of incubation with tested compounds, the cell status and the cytotoxic effect were plotted using the characteristic cell index-time profile. Growth curves were normalized to the time point of treatment. Evaluations were performed using the RTCA 1.2.1 software.

## 4. Conclusions

This study focuses on the chemical analysis of hydrolates prepared by the hydrodistillation and steam distillation of *Lavandula angustifolia* Mill., *Syzygium aromaticum* L., *Foeniculum vulgare* Mill., and *Laurus nobilis* L., as well as on assessing their antimicrobial potential on planktonic and biofilm cells. The MIC and MBC values of the tested hydrolates were recorded in the range of 0.1–12.5%. The highest antimicrobial activity was observed especially in the case of clove hydrolates. As far as we know, the antimicrobial activity of these hydrolates against especially *Arcobacter*-like bacteria has not yet been monitored. The significant antimicrobial activity of the above-mentioned hydrolates against the microorganisms tested is also described for the first time, especially after their concentration by SPE. Concentrated hydrolates also had an inhibitory effect on microbial biofilm formation. Nevertheless, an increase in biofilm activity, for example, was recorded for *A*. *lanthieri* LMG 28517, *A. butzleri* CCUG 30484 in the clove H_SD_SPE_ hydrolate, and for *A*. *thereius* LMG 24488 in the clove H_HD_SPE_ hydrolate. Moreover, concentrated hydrolates did not show any cytotoxic effect against human A549 cells. Thus, it can be concluded that hydrolates could be used as, for example, natural antimicrobial substances or food preservatives, but further testing will be needed.

## Figures and Tables

**Table 1:** Comparison of analyses of H_SD_SPE_ and H_HD_SPE_ extracts by means of peaks in chromatograms, identified compounds and three major components, *n* = 3.

Hydrolate from	Peaks in Chromatograms		Identified Compounds (Total Rel. Content)		Main Compounds (Rel. Content)	
	H_SD_SPE_	H_HD_SPE_	H_SD_SPE_	H_HD_SPE_	H_SD_SPE_	H_HD_SPE_
Lavender	186	172	48 (90.0%)	48 (93.2%)	1,8-Cineole (20.6%)	Linalool (23.2%)
					(*Z*)-Linalool furanoxide (11.9%)	1,8-Cineole (19.5%)
					α-Terpineol (10.4%)	α-Terpineol (13.0%)
Bay leaves	227	166	33 (78.0%)	33 (79.4%)	1,8-Cineol (56.4%)	1,8-Cineol (54.1%)
					4-Terpineol (6.0%)	4-Terpineol (7.1%)
					α-Terpineol (5.0%)	α-Terpineol (6.4%)
Fennel	87	68	13 (84.1%)	11 (85.1%)	Estragole (37.4%)	Estragole (33.0%)
					Fenchone 22.5%)	Fenchone (26.5%)
					*p*-Methoxy Cinnamaldehyde (7.0%)	Eugenol (5.6%)
Clove	98	80	9 (99.3%)	9 (99.3%)	Eugenol (89.1%)	Eugenol (92.7%)
					Eugenyl acetate (9.3%)	Eugenyl acetate (5.6%)
					Chavicol (0.4%)	Chavicol (0.4%)

H_HD_SPE_—hydrolate obtained by hydrodistillation and 50× concentrated using solid phase extraction (SPE); H_SD_SPE_—hydrolate obtained by steam distillation and 50× concentrated using SPE.

**Table 2:** List of compounds identified in hydrolate extracts from *Lavandula angustifolia* Mill.

CAS	Compound	RI	% of Total Peak Area	
			H_SD_SPE_	H_HD_SPE_
**Oxidized Monoterpenes**				
7392-19-0	Bois de Rose oxide/Linaloyl oxide	968	0.2	0.1
54750-70-8	(*E*)-Dehydroxy linalool oxide	987	0.1	0.1
54750-69-5	(*Z*)-Dehydroxy linalool oxide	1004	0.1	<0.1
470-67-7	1,4-Cineol	1014	<0.1	<0.1
470-82-6	1,8-Cineole	1030	20.6	19.5
5989-33-3	(*Z*)-Linalool furanoxide	1070	11.9	7.9
34995-77-2	(*E*)-Linalool furanoxide	1086	9.1	5. 9
78-70-6	Linalool	1101	7.9	23.2
29957-43-5	Hotrienol	1103	0.8	0.5
471-16-9	Sabinol	1139	0.1	0.2
76-22-2	Camphor	1144	0.4	0.5
---	Lilac aldehyde isomer (B or C)	1148	<0.1	<0.1
1786-08-9	Nerol oxide	1151	0.4	0.2
5986-38-9	(*E*)-Ocimenol	1154	<0.1	<0.1
513-20-2	Sabina ketone	1158	<0.1	<0.1
30460-92-5	Pinocarvone	1160	<0.1	<0.1
53447-47-5	Lilac aldehyde D	1163	<0.1	<0.1
14009-71-3	(*Z*)-Linalool pyranoxide	1169	1.3	1.1
39028-58-5	(*E*)-Linalool pyranoxide	1174	1.0	0.8
562-74-3	4-Terpineol	1179	1.1	1.2
500-02-7	Cryptone	1185	0.9	0.8
13741-21-4	2,6-Dimethyl-3,7-octadiene-2,6-diol	1190	5.9	2.2
98-55-5	α-Terpineol	1194	10.4	13.0
80-57-9	Verbenone	1207	0.2	0.2
1197-07-5	(*E*)-Carveol	1219	<0.1	<0.1
106-25-2	Nerol	1225	0.7	0.9
18675-34-8	Neodihydrocarveol	1231	5.9	4.9
122-03-2	Cuminaldehyde	1240	0.1	0.1
106-24-1	Geraniol	1252	1.0	2.3
51276-33-6	2,6-Dimethyl-1,7-octadien-3,6-diol	1273	1.1	0.3
536-60-7	Cumin alcohol	1291	0.4	0.3
39725-34-3	4-Hydroxy-cryptone	1322	0.2	<0.1
160152-34-1	3-Oxo-*p*-menth-1-en-7-al	1336	0.1	<0.1
7712-46-1	8-Hydroxycarvotanacetone	1427	<0.1	<0.1
26184-88-3	α-Bisabolol oxide B	1653	0.1	0.1
3790-71-4	(*2Z*,6*E*)-Farnesol	1684	0.2	0.2
Others				
111-27-3	Hexyl alcohol	872	0.2	0.2
3391-86-4	1-Octen-3-ol	981	0.2	0.2
589-98-0	3-Octanol	999	0.1	0.1
106-68-3	3-Octanone	985	0.8	0.9
---	Cymene isomer	1022	<0.1	<0.1
1073-11-6	Lavender lactone	1035	0.8	2.0
1604-28-0	6-Methyl-3,5-heptadien-2-one	1103	0.6	0.7
24903-95-5	Nopinone	1137	0.2	0.1
97-53-0	Eugenol	1351	0.5	1.3
91-64-5	Coumarin	1432	3.6	0.8
17092-92-1	Dihydroactinidiolide	1524	<0.1	<0.1
531-59-9	7-Methoxycoumarin (Hernianin)	1720	0.2	<0.1

H_HD_SPE_—hydrolate obtained by hydrodistillation and 50× concentrated using solid phase extraction (SPE); H_SD_SPE_—hydrolate obtained by steam distillation and 50× concentrated using SPE; RI—retention index relative to *n*-alkanes on SLB-5ms capillary column.

**Table 3:** List of compounds identified in hydrolate extracts from *Laurus nobilis* L.

CAS	Compound	RI	% of Total Peak Area	
			H_SD_SPE_	H_HD_SPE_
**Oxidized Monoterpenes**				
470-67-7	1,4-Cineol	1014	<0.1	<0.1
470-82-6	1,8-Cineol	1030	56.4	54.0
78-70-6	Linalool	1099	0.3	0.5
29957-43-5	Hotrienol	1101	<0.1	<0.1
36262-12-1	Dehydrosabina ketone	1120	0.1	0.1
471-16-9	Sabinol	1136	<0.1	<0.1
1786-08-9	Nerol oxide	1151	<0.1	<0.1
513-20-2	Sabina ketone	1157	0.5	0.6
30460-92-5	Pinocarvone	1160	0.4	0.3
562-74-3	4-Terpineol	1178	6.0	7.1
13741-21-4	2,6-Dimethyl-3,7-octadiene-2,6-diol	1189	0.7	0.4
98-55-5	α-Terpineol	1193	5.0	6.4
80-57-9	Verbenone	1207	<0.1	<0.1
99-48-9	Carveol	1218	0.2	0.4
18679-48-6	2-Hydroxy-1,8-cineole	1225	0.4	0.5
22626-43-3	cis-*p*-Mentha-1(7),8-diene-2-ol	1228	0.5	0.8
494-99-5	Homoveratrole	1236	<0.1	<0.1
122-03-2	Cuminaldehyde	1240	<0.1	<0.1
51276-33-6	2,6-Dimethyl-1,7-octadien-3,6-diol	1272	0.4	0.2
536-60-7	Cumin alcohol	1288	0.7	1.3
89-83-8	Thymol	1298	0.2	0.1
22539-72-6	*p*-Mentha-1,4-dien-7-ol	1328	0.2	0.2
Others				
100-52-7	Benzaldehyde	960	<0.1	<0.1
110-93-0	6-Methyl-5-hepten-2-one	985	<0.1	0.2
1073-11-6	Lavender lactone	1037	<0.1	0.1
6090-09-1	Limona ketone	1130	<0.1	<0.1
76-49-3	Bornyl acetate	1283	<0.1	<0.1
81781-24-0	1,3,3-Trimethyl-2-oxabicyclo[2.2.2]octan-5-yl acetate	1337	1.0	1.2
97-53-0	Eugenol	1350	2.2	2.4
57709-95-2	1,3,3-Trimethyl-2-oxabicyclo[2.2.2]octan-6-yl acetate	1357	0.1	0.1
121-33-5	Vanilin	1393	0.5	0.3
93-15-2	Methyleugenol	1399	1.8	1.7
17092-92-1	Dihydroactinidiolide	1524	0.2	<0.1

H_HD_SPE_—hydrolate obtained by hydrodistillation and 50× concentrated using solid phase extraction (SPE); H_SD_SPE_—hydrolate obtained by steam distillation and 50× concentrated using SPE; RI—retention index relative to *n*-alkanes on SLB-5ms capillary column.

**Table 4:** List of compounds identified in hydrolate extracts from *Foeniculum vulgare* Mill.

			% of Total Peak Area	
CAS	Compound	RI	H_SD_SPE_	H_HD_SPE_
142-62-1	Capronic acid	982	0.2	n.i.
470-82-6	1,8-Cineol	1030	2.8	3.8
122-78-1	Phenylacetaldehyde	1042	0.1	n.i.
1195-79-5	Fenchone	1086	22.5	26.5
78-70-6	Linalool	1099	0.3	0.2
76-22-2	Camphor	1144	0.7	0.9
140-67-0	Estragole	1196	37.4	33.0
99-48-9	Carveol	1218	2.6	2.3
99-49-0	Carvone	1242	2.2	2.3
123-11-5	*p*-Anisaldehyde	1253	5.2	5.5
97-53-0	Eugenol	1350	2.5	5.6
93-28-7	Eugenyl acetate	1513	0.8	1.0
1963-36-6	*p*-Methoxy Cinnamaldehyde	1567	7.1	4.0

H_HD_SPE_—hydrolate obtained by hydrodistillation and 50× concentrated using solid phase extraction (SPE); H_SD_SPE_—hydrolate obtained by steam distillation and 50× concentrated using SPE; RI—retention index relative to *n*-alkanes on SLB-5ms capillary column.

**Table 5:** List of compounds identified in hydrolate extracts from *Syzygium aromaticum* L.

CAS	Compound	RI	% of Total Peak Area	
			H_SD_SPE_	H_HD_SPE_
97-53-0	Eugenol	1360	89.1	92.7
93-28-7	Eugenyl acetate	1516	9.4	5.6
501-92-8	Chavicol	1253	0.4	0.4
121-33-5	Vanilin	1393	0.3	0.4
458-36-6	Coniferyl aldehyde	1727	0.2	0.2
119-36-8	Methyl salicylate	1189	<0.1	<0.1
87-44-5	(*E*)-β-Caryophyllene	1417	<0.1	<0.1
6753-98-6	α-Caryophyllene	1451	<0.1	<0.1
120-51-4	Benzyl benzoate	1764	<0.1	<0.1

H_HD_SPE_—hydrolate obtained by hydrodistillation and 50× concentrated using solid phase extraction (SPE); H_SD_SPE_—hydrolate obtained by steam distillation and 50× concentrated using SPE; RI—retention index relative to *n*-alkanes on SLB-5ms capillary column.

**Table 6:** Antimicrobial activity of hydrolates concentrated by SPE on *Arcobacter*-like strains—mean inhibition zones in mm (including disc 6 mm in diameter) ± standard deviation and minimal inhibitory/bactericidal concentrations in %, *n* = 4.

			*Ab* LMG 10828	*Ab* CCUG 30484	*Ab* UPa 2012/3	*Ac* CCM 7050	*Ac* UPa 2013/13	*Al* LMG 28517	*As* LMG 6621	*At* LMG 24488
**Lavender**	IZ	H_HD_SPE_	9.5 ± 0.3	8.8 ± 0.3	12.8 ± 0.3	8.3 ± 0.5	8.0 ± 0	11.3 ± 1.0	9.0 ± 0.8	11.5 ± 0.3
		H_SD_SPE_	10.8 ± 0.3	13.5 ± 0.6	10.5 ± 0.3	9.5 ± 0.9	11.5 ± 0.1	11.8 ± 0.3	10.3 ± 0.5	13.3 ± 0.9
	MIC/ MBC	H_HD_SPE_	0.4/0.8	0.8/0.8	0.8/1.6	0.8/0.8	0.8/0.8	0.8/0.8	0.4/0.4	0.8/1.6
		H_SD_SPE_	0.4/0.4	0.4/0.8	1.6/1.6	0.4/0.8	0.4/0.4	0.4/0.8	0.4/0.4	0.4/0.8
**Fennel**	IZ	H_HD_SPE_	7.8 ± 0.2	9.0 ± 0.8	10.5 ± 0.9	9.8 ± 0	8.5 ± 1.3	10.8 ± 0.5	7.8 ± 0.5	12.0 ± 0.1
		H_SD_SPE_	10.8 ± 0	10.8 ± 0.5	10.8 ± 0.9	10.0 ± 0.2	10.0 ± 0.8	11.8 ± 0	10.3 ± 0.3	11.3 ± 0.5
	MIC/ MBC	H_HD_SPE_	1.6/1.6	3.1/3.1	1.6/3.1	0.8/0.8	1.6/1.6	1.6/1.6	0.8/0.8	1.6/1.6
		H_SD_SPE_	0.8/1.6	1.6/3.1	1.6/1.6	0.4/0.8	0.8/0.8	0.8/1.6	0.4/0.4	0.8/0.8
**Bay leaves**	IZ	H_HD_SPE_	8.0 ± 0.2	9.0 ± 0.8	9.0 ± 0	9.5 ± 0.3	9.5 ± 0.3	11.3 ± 0.3	9.0 ± 0.3	10.8 ± 0
		H_SD_SPE_	10.5 ± 0.3	9.5 ± 0.6	14.0 ± 0.8	10.3 ± 0.3	10.0 ± 0.3	10.5 ± 0.6	12.5 ± 0.7	13.3 ± 0.6
	MIC/ MBC	H_HD_SPE_	1.6/1.6	1.6/1.6	1.6/3.1	0.8/1.6	0.8/1.6	1.6/3.1	1.6/3.1	1.6/1.6
		H_SD_SPE_	0.8/1.6	0.8/1.6	0.8/1.6	0.8/1.6	0.8/1.6	1.6/3.1	1.6/1.6	0.8/0.8
**Clove**	IZ	H_HD_SPE_	10.5 ± 0.3	12.0 ± 0.9	13.0 ± 0.6	10.5 ± 0.3	14.3 ± 0.5	12.0 ± 0.6	12.0 ± 0.9	14.8 ± 0.5
		H_SD_SPE_	12.5 ± 0.7	13.5 ± 0.4	14.5 ± 0.3	12.5 ± 0.3	16.5 ± 0.3	12.8 ± 0.7	11.0 ± 0.3	15.5 ± 0.7
	MIC/ MBC	H_HD_SPE_	0.1/0.1	0.2/0.2	0.8/0.8	0.2/0.2	0.4/0.4	0.4/0.4	0.2/0.2	0.4/0.4
		H_SD_SPE_	0.1/0.1	0.1/0.1	0.8/0.8	0.1/0.1	0.1/0.1	0.4/0.4	0.1/0.1	0.2/0.4
**Control**	IZ		8.0 ± 0.8	7.7 ± 0.5	7.7 ± 0.5	8.0 ± 0.8	7.0 ± 0.8	6.7 ± 0.5	8.3 ± 0.5	8.0 ± 0.8
	MIC/MBC		3.1/6.3	3.1/6.3	3.1/6.3	1.6/3.1	3.1/3.1	3.1/3.1	1.6/3.1	3.1/3.1

H_HD_SPE_—hydrolate obtained by hydrodistillation and 50× concentrated using solid phase extraction (SPE); H_SD_SPE_—hydrolate obtained by steam distillation and 50× concentrated using SPE; Control—solvent without active compounds; IZ—inhibition zone; MIC—minimal inhibitory concentration; MBC—minimal bactericidal concentration; *Ab*—*A*. *butzleri*; *Ac*—*A*. *cryaerophilus*; *Al*—*A*. *lanthieri*; *As*—*A*. *skirrowii*; *At*—*A*. *thereius*.

**Table 7:** Antimicrobial activity of hydrolates concentrated by SPE on selected microorganisms—mean inhibition zones in mm (including disc 6 mm in diameter) ± standard deviation or minimal inhibitory concentrations in %, *n* = 4.

			*Sa* CCM 4223	*Ef* CCM 4224	*Pa* CCM 3955	*Ec* CCM 3954	*Ca* CCM 8186
**Lavender**	IZ	H_HD_SPE_	8.5 ± 0.7	7.5 ± 0.6	8.5 ± 0.4	8.8 ± 0.5	6.0 ± 0
		H_SD_SPE_	8.3 ± 0.5	9.5 ± 0.3	10.5 ± 0.3	9.0 ± 0.3	6.0 ± 0
	MIC/ MBC	H_HD_SPE_	1.6/3.1	3.1/3.1	1.6/3.1	1.6/3.1	6.3/6.3
		H_SD_SPE_	1.6/3.1	1.6/3.1	1.6/1.6	1.6/3.1	6.3/6.3
**Fennel**	IZ	H_HD_SPE_	10.8 ± 0.9	9.3 ± 0.9	11.0 ± 0.8	11.3 ± 0.5	10.5 ± 0.6
		H_SD_SPE_	11.3 ± 0.7	15.0 ± 0.1	11.5 ± 0.8	11.5 ± 0.3	11.8 ± 0.7
	MIC/ MBC	H_HD_SPE_	3.1/6.3	3.1/6.3	3.1/3.1	1.6/3.1	1.6/3.1
		H_SD_SPE_	3.1/6.3	1.6/3.2	1.6/3.1	1.6/3.1	1.6/3.1
**Bay leaves**	IZ	H_HD_SPE_	9.5 ± 0.3	11.0 ± 0.8	10.3 ± 0.7	10.3 ± 0.7	9.8 ± 0.9
		H_SD_SPE_	9.5 ± 0.6	12.3 ± 0.5	11.5 ± 0.9	10.5 ± 0.9	13.3 ± 0.9
	MIC/ MBC	H_HD_SPE_	1.6/1.6	6.3/12.5	1.6/3.1	3.1/6.3	1.6/3.1
		H_SD_SPE_	1.6/1.6	6.3/6.3	3.1/6.3	1.6/3.1	0.4/0.8
**Clove**	IZ	H_HD_SPE_	15.8 ± 0.7	15.3 ± 0.3	11.3 ± 0.1	13.5 ± 0.7	14.8 ± 0.4
		H_SD_SPE_	15.5 ± 0.9	18.8 ± 0.9	12.8 ± 0.7	14.0 ± 0.8	23.5 ± 0.7
	MIC/ MBC	H_HD_SPE_	0.4/0.4	0.8/0.8	0.8/1.6	3.1/6.3	6.3/6.3
		H_SD_SPE_	0.4/0.8	0.4/0.8	0.4/0.8	1.6/3.1	1.6/3.1
**Control**	IZ		7.3 ± 0.5	9.0 ± 0.8	8.7 ± 0.5	8.0 ± 0	6.3 ± 0.5
	MIC/MBC		12.5/12.5	12.5/12.5	3.1/6.3	6.3/12.5	6.3/12.5

H_HD_SPE_—hydrolate obtained by hydrodistillation and 50× concentrated using solid phase extraction (SPE); H_SD_SPE_—hydrolate obtained by steam distillation and 50× concentrated using SPE; Control—solvent without active compounds; IZ—inhibition zone; MIC—minimal inhibitory concentration; MBC—minimal bactericidal concentration; *Sa*—*S. aureus*; *Ef*—*E. faecalis*; *Pa*—*P. aeruginosa*; *Ec*—*E. coli*; *Ca*—*C. albicans*.

**Table 8:** Testing the effectiveness of antibiotics and antifungals (mean inhibition zones in mm, including disc 6 mm in diameter ± standard deviation), *n* = 4.

	AMP	CIP	DA	E	TE	FCA
*A. butzleri* CCUG 30484	6.0 ± 0	43.5 ± 2.1	7.5 ± 0.7	37.5 ± 0.7	31.5 ± 2.1	*n.t.*
*A. butzleri* LMG 10828	6.0 ± 0	34.5 ± 0.7	6.0 ± 0	23.0 ± 0	16.0 ± 0	*n.t.*
*A. butzleri* UPa 2012/3	6.0 ± 0	39.0 ± 1.4	6.0 ± 0	29.0 ± 1.4	24.0 ± 1.4	*n.t.*
*A. cryaerophilus* CCM 7050	6.0 ± 0	25.0 ± 0	6.0 ± 0	31.5 ± 0.7	27.5 ± 0.7	*n.t.*
*A. cryaerophilus* UPa 2013/13	6.0 ± 0	36.5 ± 0.7	6.0 ± 0	30.5 ± 0.7	29.0 ± 1.4	*n.t.*
*A. lanthieri* LMG 28517	6.0 ± 0	37.0 ± 1.4	6.0 ± 0	22.0 ± 2.8	17.5 ± 0.7	*n.t.*
*A. skirrowii* LMG 6621	6.0 ± 0	41.0 ± 1.4	23.0 ± 2.8	30.0 ± 0	34.0 ± 2.8	*n.t.*
*A. thereius* LMG 24488	6.0 ± 0	32.5 ± 0.7	35.5 ± 0.7	11.0 ± 0	35.0 ± 0	*n.t.*
*S. aureus* CCM 4232	27.0 ± 0	25.5 ± 0.5	27.0 ± 1.4	28.0 ± 2.8	14.5 ± 0.7	*n.t.*
*E. faecalis* CCM 4224	13.0 ± 0	22.0 ± 0	7.0 ± 0	17.5 ± 0.7	29.0 ± 1.4	*n.t.*
*P. aeruginosa* CCM 3955	6.0 ± 0	34.0 ± 1.4	6.0 ± 0	8.5 ± 0.7	13.5 ± 0.7	*n.t.*
*E. coli* CCM 3954	6.0 ± 0	31.0 ± 1.4	6.0 ± 0	9.5 ± 0.7	20.5 ± 0.7	*n.t.*
*C. albicans* CCM 8186	*n.t.*	*n.t.*	*n.t.*	*n.t.*	*n.t.*	16.0 ± 1.4

AMP—ampicillin, CIP—ciprofloxacin, DA—clindamycin, E—erythromycin, TE—tetracycline, FCA—fluconazole, *n.t.*—not tested.
